# Validation of a Miniaturized Permeability Assay Compatible with CRISPR-Mediated Genome-Wide Screen

**DOI:** 10.1038/s41598-019-50588-0

**Published:** 2019-10-02

**Authors:** Claire Simonneau, Junning Yang, Xianguo Kong, Robert Kilker, Leonard Edelstein, Paolo Fortina, Eric Londin, Arie Horowitz

**Affiliations:** 1 Cardeza Center for Hematology Research, Thomas Jefferson University, Philadelphia, Pennsylvania USA; 2 Department of Cancer Biology, Thomas Jefferson University, Philadelphia, Pennsylvania USA; 3 Computational Medicine Center, Sidney Kimmel Medical College, Thomas Jefferson University, Philadelphia, Pennsylvania USA; 4 Department of Translational and Precision Medicine, Sapienza University, Rome, Italy; 5 Present Address: Roche Innovation Center Basel, Zürich, Switzerland; 6 Unaffiliated, Philadelphia, Pennsylvania USA

**Keywords:** Tight junctions, RNAi

## Abstract

The impermeability of the luminal endothelial cell monolayer is crucial for the normal performance of the vascular and lymphatic systems. A key to this function is the integrity of the monolayer’s intercellular junctions. The known repertoire of junction-regulating genes is incomplete. Current permeability assays are incompatible with high-throughput genome-wide screens that could identify these genes. To overcome these limitations, we designed a new permeability assay that consists of cell monolayers grown on ~150 μm microcarriers (MCs). Each MC functions as a miniature individual assay of permeability (MAP). We demonstrate that false-positive results can be minimized, and that MAP sensitivity to thrombin-induced increase in monolayer permeability is similar to the sensitivity of impedance measurement. We validated the assay by showing that the expression of single guide RNAs (sgRNAs) that target genes encoding known thrombin signaling proteins blocks effectively thrombin-induced junction disassembly, and that MAPs carrying such cells can be separated effectively by fluorescence-assisted sorting from those that carry cells expressing non-targeting sgRNAs. These results indicate that MAPs are suitable for high-throughput experimentation and for genome-wide screens for genes that mediate the disruptive effect of thrombin on endothelial cell junctions.

## Introduction

One of the new opportunities afforded by the sequencing of the human genome, as well as of many others, is the ability to run genome-wide reverse screens to identify genes that underlie diverse phenotypes. The repurposing of CRISPR/deactivated (d) Cas9 for targeting either repressive or activating modules to genomic loci provides a more versatile and specific tool than RNAi, becoming the screen method of choice^1^. In the few short years since this tool became available, it has already been applied to numerous ends, including cancer drug discovery^2^, analysis of unfolded protein response^3^, and the identification of cancer immunotherapy targets^4^. CRISPR/dCas9-mediated screens take advantage of the accurate sgRNA-dependent targeting of protein modules that either repress or activate transcription to proximal upstream and downstream regions near the transcription start site. Transcriptional activation has been achieved by various combinations of a range of modules, including NF-κB trans-activating subunit p65^5^, tetrameric VP16 transactivation domain (VP64), heat shock factor 1, the catalytic histone acetyltransferase core domain of the human E1A-associated protein p300, and the viral replication and transcription activator [reviewed in^6^]. In contrast, transcriptional repression has been produced by a single module, the Krüppel-associated box (KRAB) domain of the zinc-finger transcription factor Kox^7^. The efficacy of the regulatory dCas9-fused module is highly dependent on the selection of the sgRNA genomic targets relative to the transcription start sites, based on rules that were derived and optimized empirically^5,8^. Activation on the order of 10^3^-fold, as measured by quantifying mRNA levels of selected genes, was achieved by some of the above complexes^6^, whereas transcriptional repression measured in the same way was on the order of 10^2^-fold, and rarely 10^3^-fold^9^.

Genome-wide screens employing activating or repressive dCas9 constructs and sgRNA libraries were deployed to identify toxin or drug resistance and vulnerability-conferring genes^9–14^, genes that mediate cell immune and inflammatory responses^15–18^, and genes required for cell survival, proliferation, and metastasis^10,19–22^. The majority of screens performed to date phenotyped cell enrichment or depletion, though some studies measured the rates of differentiation^23^, cell motility^24^, and the abundance of fluorescent protein^25^. In all cases, the quantified phenotypes were individual-cell attributes. Such assays are suitable for the pooled format that, with rare exceptions^26,27^, characterizes the vast majority of the currently available sgRNA libraries, and enables a relatively facile and economical quantification of phenotypes. Though effective and fruitful, the current limitation to individual-cell functional assays leaves out collective cell functions which underlie physiologically essential processes. Here we validate a new functional assay we designed to facilitate adaptation of the CRISPR-mediated screen for the identification of genes that regulate the permeability of endothelial cell (EC) monolayers. The integrity of this monolayer is critical to the normal function of the vascular system because it confers the barrier function of the vessels. Since administration of thrombin produces large gaps in EC monolayers that can be detected with relative ease, we chose to validate the assay by testing if sgRNAs from an existing repressive CRISPR library^8^ designed to knockdown the transcription of thrombin signaling pathway genes, become enriched in unresponsive cells. Thrombin-induced permeability is not manifest in vertebrates under normal conditions, but does occur under trauma, sepsis, and other inflammation-inducing conditions in both animal models^28–30^ and patients^31,32^.

Currently available permeability assays are not compatible with the requirements of genome-wide screens because they cannot accommodate the vast number of screened samples, and because their multi-well format is unsuitable for the pooled sgRNA libraries used in practically all screens. To our knowledge, the highest sampling capacity currently available for the measurement of paracellular permeability is provided by the 96-well plate filter insert assay format. A comprehensive screen of the human genome by 10 sgRNAs and 5 technical replicates per gene using this assay would require approximately 10^4^ multi-well plates, a number that is beyond the capacity of most research laboratories. To design a more feasible assay, we took advantage of the MC-based cell culture technique developed to maximize the number of adherent cells grown per unit volume of medium. Typically, MCs consist of spherical beads made of cross-linked polymers of a diameter in the range of 100–200 μm. Commercially available MCs come in a variety of polymeric matrices and surface treatments or coatings^33^. MCs carrying EC monolayers had been used to study the effect of various agonists on cell monolayer permeability by measuring the absorbance of dye into MCs, or in a chromatographic format by measuring the elution time of a dye^34–37^.

## Results

### EC monolayers grown on single MCs serve as individual permeability assays

**Figure 1:**
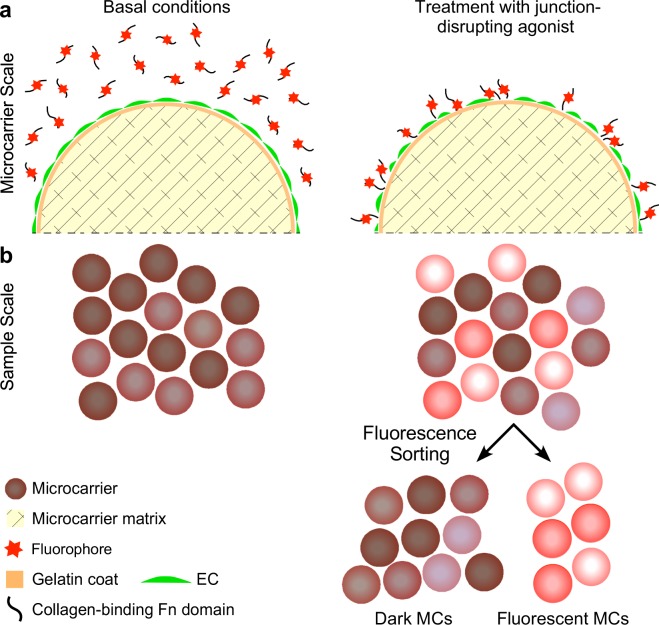
Scheme of the MAP design. (**a**) Gelatin-coated MCs composed of cross-linked dextran carry a confluent EC monolayer (green, to designate calcein-loaded ECs), incubated in medium containing the collagen-binding proteolytic fragment of fibronectin conjugated to a fluorophore (FN_cf_). Once treated by a junction-disrupting agonist (thrombin, in this study), FN_cf_ binds to the exposed gelatin surface between cells whose junctions disassembled in response to the agonist. (**b**) MCs carrying untreated ECs bind a minimal amount of FN_cf_. MCs carrying agonist-treated ECs bind varying amounts of FN_cf,_, depending on the identity of the sgRNA expressed by the clonal cell population on each MC. MCs carrying ECs that express sgRNAs targeting genes that encode proteins required for the induction of the disassembly of cell-cell junctions bind a low amount FN_cf,_, similar to untreated MCs. ECs expressing sgRNAs that are unrelated to the signaling pathway of the junction-disrupting agonist respond by disassembling their junctions. FN_cf_ binds to the gelatin surface exposed between the responsive ECs, rendering the MCs that carry these cells fluorescent. The fluorescent MCs are separated from the dark MCs by fluorescence-assisted sorting. The gates of the sorting machine can be set up to capture any group of interest in this population, based on MC fluorescence amplitude.

We sought an *in vitro* permeability assay that would be compatible with the high throughput format required for genome-wide screens to interrogate signaling pathways that regulate the integrity of endothelial cell junctions. In the new assay, each MC is treated as an individual MAP. This facilitates high throughput testing of a large number of samples in a relatively small volume of growth medium (approximately 6.2 × 10^5^ MCs per 100 mL). We envisioned that ECs will be transduced by repressive (CRISPRi) sgRNA libraries and grown as clonal populations on each MC. When treated by agonists that disrupt cell junctions, e.g. vascular endothelial growth factor (VEGF) or thrombin, the junctions among ECs expressing sgRNAs targeting genes required the for cellular response to the administered agonist would fail to disassemble. To distinguish between MCs carrying responsive or non-responsive EC monolayers, we selected a probe that would bind to the MC surface exposed through the gaps between responsive cells. Since the MC type that supported EC growth optimally is coated with gelatin, we selected the collagen-binding fluorescently-conjugated fragment of fibronectin (FN_c_)^38^ as a probe because of its high binding affinity to gelatin^39^. The fluorescence of MAPs carrying responsive EC monolayers would increase upon thrombin treatment because of the binding of fluorescently conjugated FN_c_ (FN_cf_) to the newly formed gaps between ECs. The darker MAPs that carry non-responsive monolayers would then be separated from the brighter ones by fluorescence-assisted sorting (Fig. 1).

### Thrombin increases the permeability of telomerase-immortalized human primary EC monolayers

**Figure 2:**
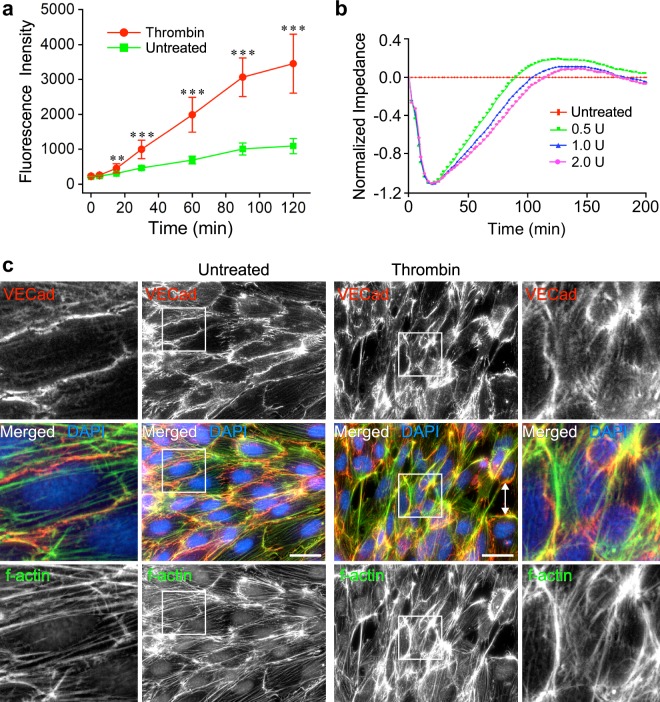
Thrombin increases the paracellular permeability of TIME cell monolayers. (**a**) The permeability of TIME cell confluent monolayers grown on filter inserts to 2000 kDa FITC-dextran increased in response to treatment by 2.0 U thrombin applied at t = 0 min for the duration of the experiment, as indicated by the augmentation of the fluorescence signal from the bottom wells (mean ± SD, n = 9, **P < 0.01, ***P < 0.001). (**b**) The impedance of TIME cell monolayers grown on electronic 16-well plates fell sharply after treatment by the indicated dosages of thrombin (applied as above), indicating that the integrity of the monolayer deteriorated. (**c**) Epifluorescence images of confluent TIME cells in monolayers grown on gelatin-coated coverslips. Intercellular junctions disassembled upon treatment by 2 U/mL thrombin (Thr), as indicated by the change in the VEcad pattern. The cells formed thicker stress fibers and detached from each other. Magnified images of regions surrounded by white frames are shown on the left or right side of each image. The double-pointed white arrow marks a gap between thrombin-treated cells. Scale bars, 50 μm.

Among a variety of known permeability factors, thrombin induces relatively large openings between confluent ECs^40^ cultured on the same type of MCs used in this study^35^. To gauge thrombin’s effects on monolayers of telomerase-immortalized (human dermal) microvascular endothelial (TIME) cells, we measured its impact on permeability by two approaches. In the first, we measured the penetration of a fluorescently conjugated dextran probe through an EC monolayer. In the second, we measured thrombin-induced change in monolayer impedance. Both approaches yielded large changes in the measured attributes, indicating substantial increases in monolayer permeability. In the first type of assay, the fluorescence of the probe in the lower chamber of the assay almost plateaued after a more than a 3-fold increase (Fig. 2a). In the second assay, thrombin concentrations of 0.5, 1.0, and 2.0 U/mL reduced the impedance of TIME cell monolayers by approximately one unit (Fig. 2b). To visualize the structural effects of thrombin on the EC monolayer, we immunolabeled the cells to track the localization of vascular endothelial cadherin (VEcad) and used phalloidin to observe thrombin-induced changes in the actin cytoskeleton. Thrombin induced the opening of large gaps that reached a width of approximately 10 μm and formation of denser and thicker stress fibers (Fig. 2c), similar to previous observations^41^.

### Single TIME cells grown to confluence on gelatin-coated MCs

**Figure 3:**
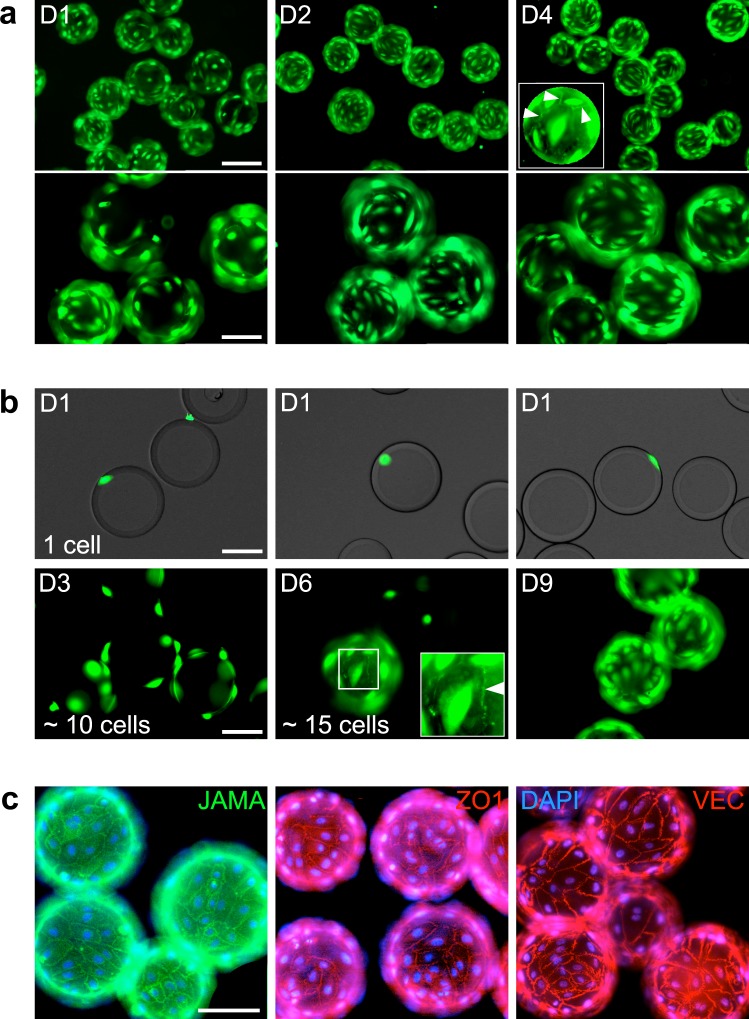
TIME cells form a clonal confluent cobble-stone monolayer on MCs. (**a**) Images of calcein AM-stained TIME cells seeded on MCs at a density of 1.5 × 10^4^ cells/cm^2^ surface area, grown for the indicated durations in spinner flasks. The cells reached confluence after 4 days and formed a tightly sealed monolayer (see cell junctions marked by white arrowheads in the inset). Scale bars, 200 (top row) or 100 (bottom row) μm. (**b**) When initiated from a single TIME cell per MC, a confluent monolayer formed in 9 days. The inset visualizes intercellular junctions marked by a white arrowhead. Scale bar, 100 μm. (**c**) MCs coated by confluent TIME cell monolayers immunolabeled with the indicated antibodies. Scale bar, 100 μm.

We determined that gelatin-coated MCs consisting of cross-linked dextran matrix are optimal for TIME cell growth. To observe monolayer integrity, we stained the cells with calcein, visualizing both the cell body and intercellular junctions (Fig. 3a). In a genome-wide screen, cells grown on each MC should express a single sgRNA to avoid mixed effects of silencing the expression of potentially antagonistic genes or the additive effects of potentially synergistic genes. Consequently, the TIME cell monolayer on each MC has to evolve from a single cell in order to generate a monoclonal population. To achieve a one-to-one cell-MC ratio, we used a seeding ratio of one cell per two MCs. Single TIME cell reached confluence in nine days, forming tightly sealed monolayers similar to cells grown from a larger initial number (Fig. 3b).

### TIME cells grown on MCs form intact tight and adherens junctions

Formation and maintenance of a cobblestone monolayer with integral intercellular junctions is essential for the use of MAPs as permeability assay samples. To find if junction-specific proteins are present at TIME cell intercellular junctions, we probed the cells for the tight junction single-pass transmembrane protein JAMA, and for the adaptor protein ZO1, which bind each other^42^. Both proteins were abundantly present at the borders of all the cells that were visible in the field of view, appearing as narrow undisrupted bands (Fig. 3c). The adherens junction marker VEcad was similarly abundant and patterned (ibid.). We conclude that the intercellular junctions formed by MAPs do not differ from junctions formed by the same (Fig. 2c) or other^43^ ECs on flat surfaces.

### Thrombin increases attachment of FNc to gelatin-coated surfaces

To assess the performance of FN_cf_ as a probe (Appendix SI, Fig. S1a), we tested if the extent of its binding to a gelatin-coated substrate decreases as EC confluence increases. The fluorescence signal of the probe attached to a glass coverslip harboring a confluent TIME cell monolayer was miniscule in comparison to the signal emanating from a sub-confluent monolayer (Appendix SI, Fig. S1b). Thrombin treatment of confluent TIME cell monolayers generated large gaps between adjacent cells (Appendix SI, Fig. S1c). Bare gelatin-coated MCs bound FN_cf_ avidly, whereas MCs coated by a sub-confluent EC monolayer bound only a fraction of the latter (Appendix SI, Fig. S1d).

### Likelihood of false discovery is reduced by removal of non-confluent MCs

**Figure 4:**
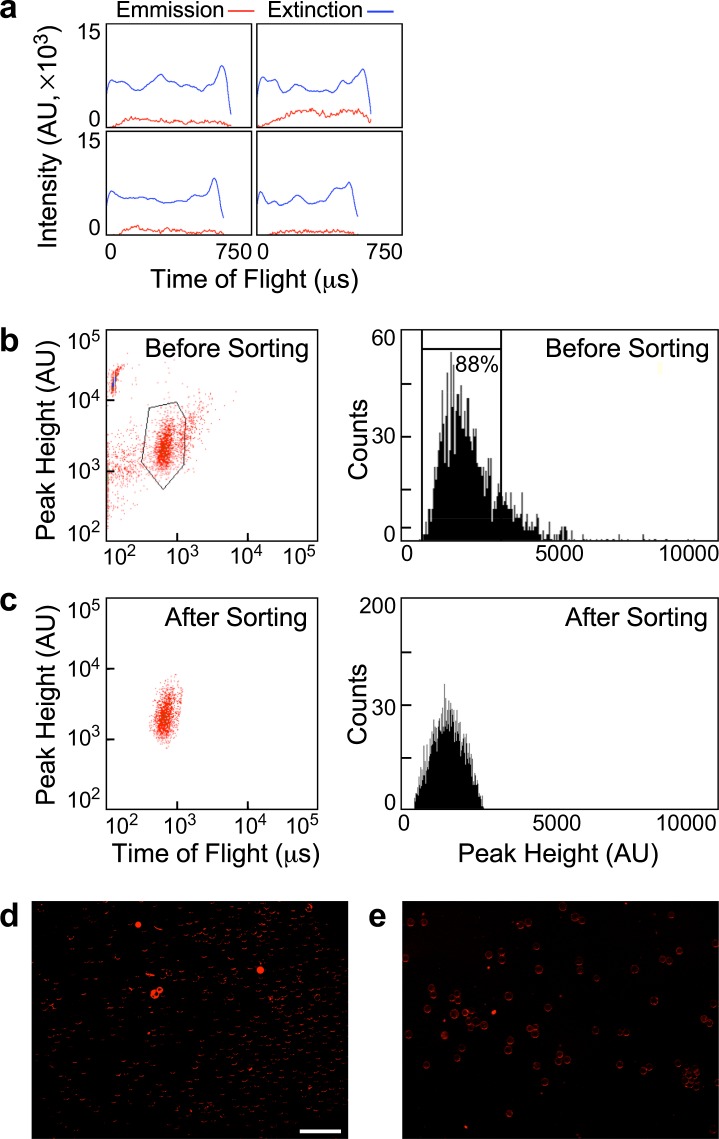
Binding of FN_cf_ excludes non-confluent MAPs. (**a**) Gallery of emission and extinction intensity profiles of individual MAPs. (**b**) Left side: density plot of MAP extinction per time-of-flight of an untreated MAP population that was incubated with FN_cf_ prior to sorting; right side: histogram of the MAP population shown in the density plot on its left; the gate is placed at approximately 2-fold intensity of the median peak MAP fluorescence amplitude. (**c**) Left side: density plot of the MAP population gated above; right side: histogram of the MAP population shown in the density plot on its left. (**d**) Image of a group of presorted MAPs showing the fluorescence of MC-bound FN_cf_. (**e**) A similar image of a group of sorted MAPs (scale bar, 1 mm).

Though the majority of TIME cell monolayers reached confluence even when grown from a single cell per MC, it is practically impossible to ensure that the surface of all MCs is fully coated by cells. Elimination of MCs that have residual exposed surface is essential because the FN_cf_ probe would bind to the exposed surface, causing misidentification of the cells as intransigent to the sgRNA they had been transduced with, and the overlooking potentially relevant genes. To eliminate sub-confluent MCs from the population, we sorted MAPs that were incubated with FN_cf_ in the absence of an agonist. MAP extinction profiles were frequently uniform across the imaged face of the MC but peaked at the edges of individual MAPs (Fig. 4a). The peaks corresponded to the gelatin and TIME cell layers. The initial gating of the MAP population was chosen to exclude debris or MAP clusters (Fig. 4b). The residual MAP population was gated again to remove MAPs whose peak fluorescence intensity was at least two-fold higher than the intensity of the highest counts (ibid.). We assumed this would eliminate most of the non-confluent MAPs. The removed fraction amounted to 12 percent of the MAP population. Consequently, the peak-height histogram of the residual MAPs was confined to a narrow range (Fig. 4c). Visual inspection of samples from the initial and residual populations confirmed the presence of exceptionally bright MAPs in the former (Fig. 4d), but not in the latter (Fig. 4e).

### sgRNAs repress target gene expression, protein abundance, and cell monolayer response to thrombin

**Figure 5:**
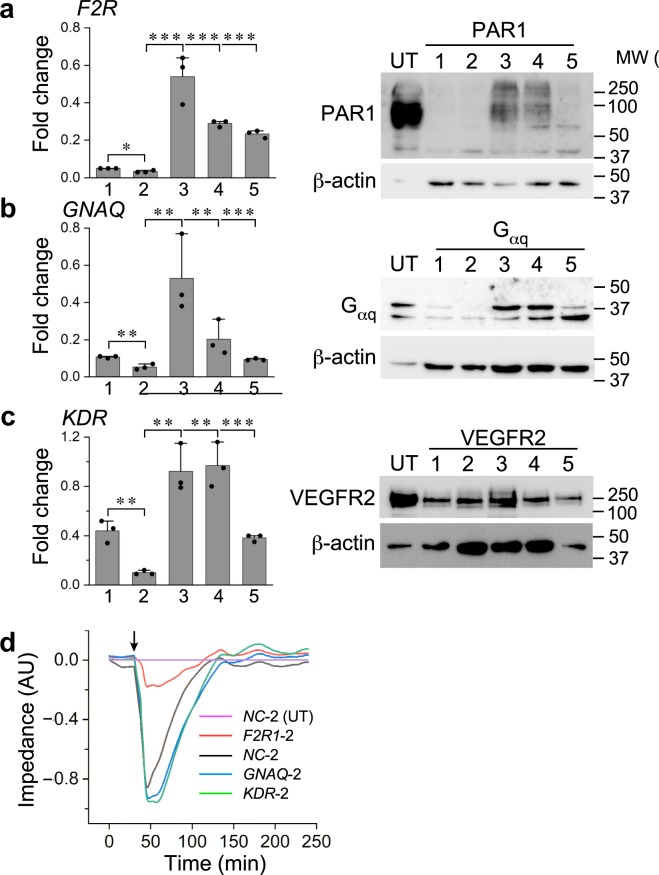
Highly effective sgRNAs reduce substantially the expression and abundance of the targeted gene and the encoded protein, respectively. (**a**) Quantification of qRT-PCR measurements of the expression levels of *F2R* in TIME cells transduced by each one of the five *F2R*-targeting CRISPRi sgRNAs. The immunoblot on the right shows PAR1 abundance in TIME cells transduced by the same sgRNAs. The first lane on the left was loaded with lysate of TIME untransduced (UT) cells. β-actin was used as a loading control for all immunoblots. (**b**,**c**) The same measurements as above for the sgRNAs that target *GNAQ* or *KDR*, and the corresponding immunoblots (**a**–**c**: mean ± SD, n = 3, *P < 0.05, **P < 0.01, ***P < 0.001). (**d**) Impedance traces of TIME cells expressing each of the indicated sgRNAs. Thrombin was applied to all the cell groups at the arrow-marked time point. The traces were normalized relative to the impedance of untreated (UT) cells expressing a negative control (NC-2) sgRNA.

To test if the MC-based permeability assay can identify genes that affect EC response to thrombin, we selected sgRNAs that target *F2R*, the gene that encodes the main thrombin receptor in ECs^44^, proteinase-activated receptor (PAR) 1, as a positive control. We chose *KDR*, the gene that encodes VEGF receptor 2, the main VEGF receptor in blood vessel ECs^45^, as a negative control. We included sgRNAs that target *GNAQ*, a gene encoding Gα_q_, a trimeric GTPase. Gα_q_ mobilizes calcium by activating phospholipase C-β, but, unlike Gα_12/13_, there is no consensus in regard to its ability to increase EC monolayer permeability^46,47^. We selected it, therefore, as a gene of unknown effect on TIME cell response to thrombin. Before testing the effects of the selected sgRNAs on EC barrier function, we validated the presumed capacity of each of the five individual sgRNAs designed to target these genes^8^ by measuring how effectively they repressed the transcription of their target genes and, consequently, reduced the abundance of the proteins these genes encode. Using quantitative real-time polymerase chain reaction (qRT-PCR), we detected a large variability among the repressive effects within each set of five sgRNAs. The most effective sgRNA repressed *F2R* transcription to approximately 6 percent relative to its expression level in TIME cells treated by non-targeting sgRNAs (Fig. 5a). Immunoblotting indicated that PAR1 abundance in each cell group expressing a single sgRNA fit closely the qRT-PCR expression pattern of *F2R* once the weight of each band was normalized relative to the weight of the corresponding loading-control β-actin band (Fig. S2a). The sgRNA-specific expression levels of *GNAQ* and *KDR* fit well the abundances of Gα_q_ and VEGFR2 (Fig. 5b,c), confirming that two to three sgRNAs out of each set of five repressed their target gene by at least 60 percent.

To test the functional effect of sgRNA expression, we measured the impedance of TIME cell populations expressing each of the most repressive sgRNAs of *F2R*, *GNAQ*, and *KDR* before and after thrombin application. The impedance of TIME cells expressing *GNAQ*, *KDR*, and validated^8^ negative-control (NC) sgRNAs fell by close to one impedance unit (Fig. 5d). The impedance of TIME cells expressing *F2R* sgRNAs, on the other hand, fell only by 0.2 units.

### Fluorescence-assisted sorting separates MAPs carrying thrombin-treated and untreated TIME cells

**Figure 6:**
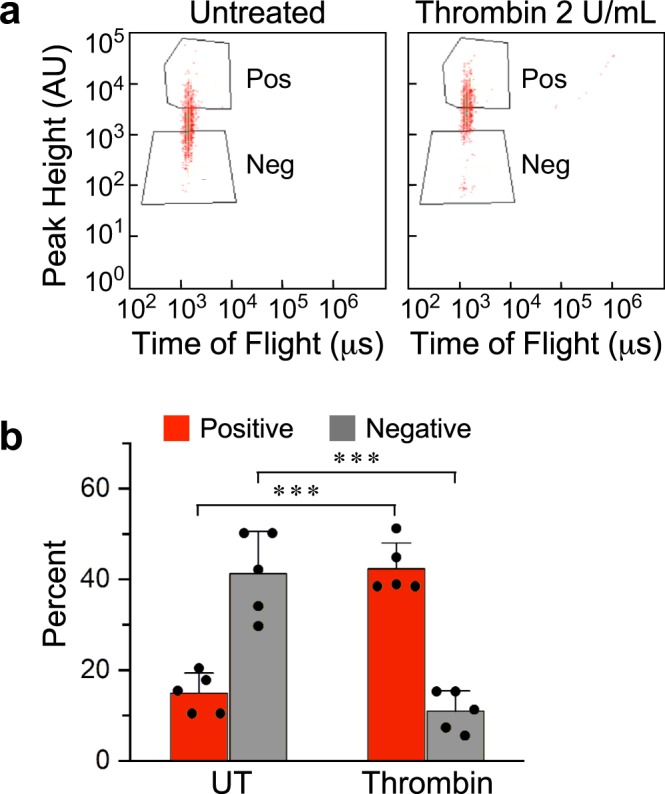
Dark or fluorescent MCs are over-abundant in untreated or in thrombin-treated populations, respectively. (**a**) Confluent TIME cells grown on MCs were either untreated or treated by 2 U/mL thrombin for 30 min, followed by 5 min incubation with 1 μg/mL FN_cf_. The MCs were sorted according to the indicated gates into positive (Pos) and negative (Neg) subgroups. (**b**) Histogram showing the negative and positive fractions of untreated or thrombin-treated MAP populations gated shown in panel a (mean ± SD, n = 5, ***P < 0.001).

Execution of a genome-wide screen requires physical separation between the phenotypes that ensue during the functional assay. In the current study, these are thrombin-responsive and non-responsive MAPs. FN_cf_ would enable separation by fluorescence-assisted sorting. After thrombin treatment and before sorting, we fixed the MC-attached TIME cells by EtOH, an optimal fixative for the preservation of nucleic acids^48^, to prevent changes in cell and junction morphology. Due to heterogeneity in cell response to thrombin, the light-intensity distribution among the sorted MAPs was continuous over an interval of intensity values, rather than binary (Fig. 6a). To produce two distinct sorted populations, we defined two gates, one for the low-responding MCs, referred to as ‘negative’, and another for the high-responders that we refer to as ‘positive’ (ibid.). The fraction of untreated negative MAPs fell from approximately 40 percent to only 10 percent of the thrombin-treated MC population because of the upward shift in MAP fluorescence intensity (Fig. 6b). Complementarily, the percentage of positive MCs increased from approximately 15 to 40 percent of the treated MC population. These measurements demonstrate a robust and reproducible response to thrombin that can be readily resolved into positive and negative populations. A portion of approximately 45 percent of the total MC population, encompassing the medium range of response, remained between the upper and lower gates. This sub-population is least likely to contain MCs carrying cells that express the most repressive sgRNAs. Sidestepping this population is not likely, therefore, to preclude the identification of genes that have major roles in mediating the disassembly of cell junctions in response to thrombin.

### MAP sensitivity to *F2R* knockdown is similar to the sensitivity of impedance measurement

**Figure 7:**
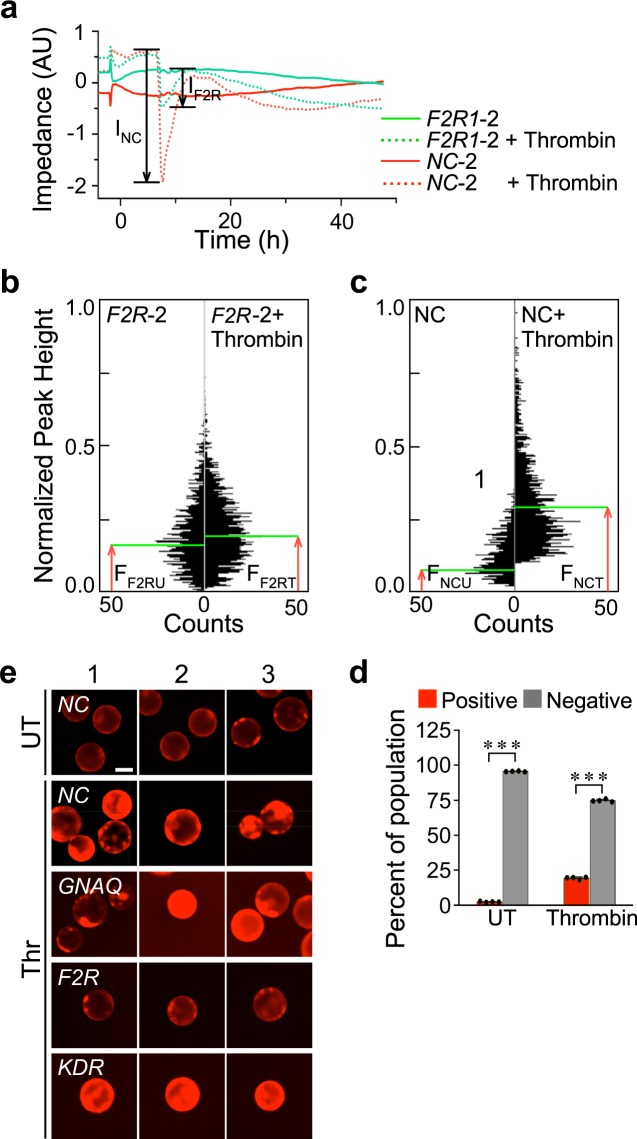
*F2R* sgRNA suppresses thrombin-induced hyperpermeability. (**a**) Time course of impedance measurements of thrombin-treated TIME cells normalized relative to the impedance of untreated (UT) cells expressing a negative control (NC-2) sgRNA. (**b**) Density plot and gating of the peak-height fluorescence of MAPs carrying cells transduced by *F2R*-targeting sgRNA; righthand panel: counts per peak-height and gate of the MAP population shown in the density plot. (**c**) Counts per peak-height fluorescence of sorted MAPs that carry either untreated or thrombin-treated cells transduced by *F2R*-targeting sgRNA. (**d**) Effects of thrombin treatment on MAP separation into positive and negative fractions (mean ± SD, n = 4, ***P < 0.001). (**e**) Galleries of triplicate fluorescence images of MAPs carrying cells transduced by the indicated sgRNAs under the specified conditions. Scale bar, 100 μm.

We chose impedance measurement as a benchmark for assay sensitivity because this technique is more sensitive than that of probe flux through cell monolayers cultured on permeable substrates^49^, the main alternative technique. We tested the sensitivity of impedance measurement as a detector of sgRNA-mediated knockdown by comparing the thrombin response of TIME cells transduced by the most effective *F2R*-targeting sgRNA, *F2R-*2, to cells transduced by a negative-control sgRNA. Whereas the impedance of cells transduced by the latter sgRNA fell by 2.6 units (denoted as I_NC_ in Fig. 7a), the impedance of TIME cells transduced by *F2R*-2 fell only by 0.75 unit (I_F2R_).

As a first step in the test of MAP sensitivity to *F2R* knockdown, we repeated the procedure described above for the removal of particles that were not whole single MAPs and of non-confluent single MAPs (Fig. 4b). The mean peak fluorescence intensity of thrombin-treated MAPs carrying cells transduced by the most effective *F2R*-targeting sgRNA (denoted as F_F2RT_ in Fig. 7b) was higher by approximately 18 percent than that of the same type of untreated MAPs (F_F2RU_). On the other hand, MAPs carrying cells transduced by a mixture of three negative control sgRNAs responded to thrombin by a four-fold increase in mean fluorescence intensity compared to an untreated group (compare F_NCT_ to F_NCU_ in Fig. 7c). Because impedance measurement sensitivity is a dimensionless variable given by the ratio between I_NC_ and I_F2R_ (Fig. 7a), we defined MAP sensitivity as the ratio between the response of cells which express a mixture of three negative control sgRNAs and cells that express *F2R*-2, i.e. the ratio between F_NCT_−F_NCU_ (Fig. 7b) and F_F2RU_−F_F2RT_ (Fig. 7c). The respective sensitivities were 3.5 and 7.6, indicating that MAP sensitivity was comparable or better than the sensitivity of impedance measurement for detecting the repressive effect of the *F2R*-2 targeting sgRNA.

### *F2R* sgRNA suppresses thrombin-induced hyperpermeability

All the sgRNAs tested above repress, albeit to varying degrees, the expression of their target genes. Therefore, we gauged the combined effect of these sgRNAs on MAP permeability by comparing the untreated and thrombin-treated fractions of positive and negative MAPs sorted from a mixed population of cells that express on each MAP one of the 15 sgRNAs employed in the study (five each for *F2R*, *GNAQ*, and *KDR*). The sorting of untreated MAPs (Fig. 7d) almost abolished the population of untreated positive MAPs, while the sorting of thrombin-treated MAPs reversed the distribution of thrombin-treated MAPs shown in Fig. 6b. The former was more than halved, and the latter increased by close to seven-fold. These large effects demonstrate the repressive efficacy of each gene-specific group of five sgRNAs. To visualize the individual effect of the most repressive *F2R*, *GNAQ* and *KDR*-targeted sgRNAs on MAP permeability in comparison to three validated NC sgRNAs^8^, we imaged the fluorescence of thrombin-treated MAPs expressing each of these sgRNAs. Cells expressing either *GNAQ*, or *KDR*-targeted sgRNAs responded to thrombin similarly to negative-control sgRNA-expressing MAPs (Fig. 7e). In contrast, the response of MAPs carrying cells that expressed the most effective *F2R* sgRNA was similar to that of MAPs carrying untreated cells that expressed one of the negative control sgRNAs (top row images in Fig. 7e). These results are consistent with the functional effects of the sgRNAs in Fig. 5d. The low fluorescence intensity emitted by MAPs carrying cells that express a *F2R* sgRNA indicates that thrombin treatment did not generate an artifactual signal caused by cell detachment from the MC surface. Furthermore, an assay with MAPs that express a repressive sgRNA library requires sorting of MCs that emit weak light signals. Therefore, artifactually bright MCs would not cause false gene identification.

## Discussion

Permeability is not amenable to existing functional assays because it is inherently a collective cell function. The new assay we designed repurposes a pre-existing device – MCs that had been developed over fifty years ago to increase the surface available for the growth of adherent cells^50^. MCs had already been used, in fact, to measure EC monolayer permeability, albeit in bulk format^35^. In contrast, the new assay deploys each cell-coated MC as a single sample, thus taking advantage of its small diameter to pack as many as 6000 MCs per mL of medium. At this sample concentration, 166 mL of growth medium would contain 10^6^ MCs. As estimated above, this is a sufficiently large number of samples for performing a genome-wide screen with this functional assay.

Screens based on cell growth can measure a continuum of quantitative phenotypes by counting the numbers of cells expressing sgRNAs of interest^51^. These measurements can be used for further analysis of the targets identified in the first screen, such as construction of genetic interaction (GI) maps^52^. Though the phenotypes of the MAPs (i.e., their fluorescence intensities), vary continuously within a certain range, the sorting converts that range into an either ‘positive’ or ‘negative’ binary output, precluding the attribution of a continuous range of quantitative phenotypes to each sample and of further analysis. This drawback can be overcome, however, by performing a set of assays where the position of the gate is changed incrementally so that a larger range of phenotypes is sampled. Replicating the assay is straightforward because it can be run in as little as 30 min using a volume of less than 200 mL MCs in growth medium.

In addition to compactness, the format of the assay facilitates physical separation between samples that are either responsive or refractory to the stimulus by using the phenotype as a sorting input. The separation is crucial for the identification of the genes that produce the measured phenotype. Counting the number of cells on the MCs, and determining if the cell monolayer reached confluence are straightforward because the cells on the MC surface can be readily visualized by dyes, or other imaging modalities^35,53^. The visibility enables growth of a clonal cell population from a single cell per MAP and verification that the cell monolayer coating the MAPs is confluent. This prevents spurious signal as a result of probe binding to the exposed surface of sub-confluent MCs. The duration of the assay, i.e. the time necessary for eliciting a measurable phenotype, depends on the nature of the agonist, but it is not likely to allow sufficient time for the emergence of multiple phenotypes, such as change in cell density. This narrows the scope of the assay to its intended purpose of measuring intercellular permeability and prevents interference from other potential effects of the agonists which are often pleiotropic, such as VEGF.

As is standard practice in pioneering proof-of-principle studies, we favored permissive experimental conditions. Accordingly, we chose an agonist that induces relatively large intercellular gaps that did not strain the detection limits of our probe. Admittedly, other agonists produce subtler remodeling of intercellular junctions, requiring higher sensitivity for distinguishing between the signals emitted by monolayers of quiescent versus stimulated ECs. If the agonist induces sufficiently large openings between adjacent cells to permit binding of the probe to the MC surface, and if the binding affinity between the probe and the surface is sufficiently high, our approach should be feasible for such agonists. VEGF, an obvious agonist of interest, generates gaps on the order of several micrometers^54^. Its signaling pathway should be amenable, therefore, to MAP screening. The MAP configuration limits to some extent the range of testable conditions because it does not simulate abluminally-acting agonists, including, in some cases, VEGR2-downstream effects^55^. Genes that are required for cell proliferation and for adhesion to gelatin will not be represented in the screened cell population because cells that express sgRNAs that target these genes will be lost during cell growth on MCs. It is unlikely, however, that such genes would be directly involved in regulating intercellular junction integrity.

## Materials and Methods

### Endothelial cell culture

TIME cells (American Type Cell Culture) were grown at 37 °C in a humidified 5 percent CO_2_ incubator in Vascular Cell Basal Medium supplemented with Microvascular Endothelial growth Kit-VEGF (American Type Cell Culture) and 12.5 μg/mL blasticidin (ThermoFisher Scientific).

### Immunofluorescence

TIME cells grown to confluence on gelatin-coated round 12-mm glass coverslips (GG-12-Gelatin, Neuvitro) were treated with 2 U/mL thrombin (human α-thrombin, Enzyme Research Laboratories) for 30 min at 37 °C and 5 percent CO_2_, or with PBS as a negative control. After washing in ice-cold PBS, fixation in 4 percent paraformaldehyde/PBS for 30 min at 23 °C, and permeabilization in 0.1 percent Triton X-100 (Sigma-Aldrich) in PBS for 5 min, cells were incubated in a blocking solution of 5 percent BSA (Sigma-Aldrich) in PBS for 1 h. Immunolabeling by primary antibodies diluted according to the manufacturer’s instructions in 0.1 percent Triton X-100 and 1 percent BSA in PBS overnight at 4 °C was followed by 3 × PBS washes and incubation with 2.5 μg/mL fluorescently-conjugated secondary antibodies in PBS for 30 min at 23 °C. After 3 × PBS washes, coverslips were mounted on glass slides (ProLong Gold Antifade Mountant, ThermoFisher Scientific). Images of TIME cells grown either as a two-dimensional culture, or on MCs, were acquired by epifluorescence (EVOS FL Auto Cell Imaging System, ThermoFisher Scientific) or by laser-scanning confocal microscopy (Nikon A1R+).

### Immunoblotting

Cells were scraped off plates, extracted in RIPA lysis buffer (25 mM Tris-HCl pH 7.6, 150 mM NaCl, 1 percent NP-40, 1 percent sodium deoxycholate, 0.1 percent SDS; ThermoFisher Scientific) supplemented with phosphatase (Fisher Scientific) and protease (Sigma-Aldrich) inhibitor cocktails, and clarified by centrifugation (20,000 g, 10 min). Total protein concentration was determined by BCA protein assay (ThermoFisher Scientific). Equal amounts of total protein were separated by 10 percent SDS-PAGE and transferred to a PVDF membrane. Membranes were blocked by 5 percent casein in TBS with 0.1 percent Tween 20 (Sigma-Aldrich), probed with the indicated primary antibodies, and incubated with HRP-conjugated secondary antibodies for 1 h at 23 °C. Protein-antibody complexes were visualized by chemiluminescence (SuperSignal™ West Pico PLUS, ThermoFisher Scientific) and imaged by a charge-coupled device camera (Chemidoc Imaging System, BioRad). Band densitometry was measured with the Gels submenu of ImageJ version 2.0.0.

### Impedance measurement

Impedance was measured on xCELLigence Real-Time Cell Analyzer (ACEA Biosciences) according to the manufacturer’s instructions. Briefly, 30000 TIME cells were seeded in 200 μL growth medium per well of an electronic 16-well plate (E-Plate View 16, ACEA Biosciences) for 30 min at 23 °C to allow cell sedimentation. The cells were grown to confluence at 37 °C, 5 percent CO_2_. On the day of the experiment, cells were treated by thrombin or by the same volume of carrier (PBS), and placed in the analyzer for continuous recording of impedance. The relative impedance output of the instrument was expressed as (Z_i_-Z_0_)/15 Ω, where Z_i_ is the impedance at the i^th^ time point, and Z_0_ is the initial impedance before agonist addition.

### Macromolecular permeability

TIME cells seeded at 1 × 10^5^ per filter insert (0.4 μm pores, 6.4 mm diameter, polyethylene terephthalate membrane, Transwell®, Corning) were grown for 72 h in 24-well plates (Transwell®, Corning) filled with 800 μL growth medium per well. The cells on the inserts were supplemented at the same time by either 2 U/mL thrombin or by carrier, and by 1 μg/mL fluorescein isothiocyanate (FITC)-dextran (2000 kDa, Sigma-Aldrich) that were added to the upper chamber. 10-μL medium samples were removed after 0, 5, 15, 30, 60, 90 and 120 min from the lower compartment, diluted in 90 μL of PBS, and transferred to a 96-well plate with a clear bottom. Fluorescence in each well was measured at 485 nm excitation and 535 nm emission (VICTOR2, PerkinElmer).

### TIME cell culture on MCs in spinner flasks

Cytodex-3 MCs (GE Health Sciences) were hydrated in PBS at 50 mL/g for 3 h at 23 °C, washed twice in PBS, and autoclaved (121 °C, 20 min). Aliquots of 92 mg cytodex-3 MCs were readied for cell growth by incubation overnight at 37 °C, 5 percent CO_2_, and stirred at 35 rpm (Advanced Multi-Position Stirrer, Troemner) in 50 mL growth medium in siliconized (Sigmacote, Sigma-Aldrich) spinner flasks (Bellco Glass, model 1965–95010) equipped with a glass-ball plunger. TIME cells grown to 80 percent confluence in T75 flasks were briefly detached with 0.05 percent trypsin, 0.02 percent EDTA (American Type Cell Culture). The cells were resuspended in growth medium supplemented with 15 percent FBS and 4.5 g/L glucose. MCs were inoculated with 6 × 10^6^ cells in 50 mL growth medium to achieve a density of 24000 cells/cm^2^. The MC culture was incubated at 37 °C, 5 percent CO_2_, stirred intermittently at 35 rpm for 1 min once every 15 min for a total of 1 hour, and then stirred continuously at the same speed. Medium was replenished once every two days. Cells reached confluence in 3–5 days. In order to seed a single cell per MC, the above aliquot of MCs was inoculated with 1.5 × 10^5^ cells to achieve a density of 600 cells/cm^2^. To monitor cell growth on MCs, a small MC sample was removed every day, treated with 2 μM of Calcein AM (ThermoFisher Scientific), and visualized by epifluorescence microscopy. Under these conditions, cells reached confluence in 9 days.

### Detection of barrier disruption by fluorophore-conjugated fragment of fibronectin

To detect disruption of confluent TIME cell monolayers on MCs, we used the 45 kDa collagen-binding of fibronectin (FN_c_) purified from human plasma (Sigma-Aldrich) conjugated to Dylight 550 with a labeling kit (ThermoFisher Scientific). The cells were grown to confluence either on gelatin-coated round 12-mm glass coverslips (GG-12-Gelatin, Neuvitro), or in spinner flasks spun at 35 rpm at 37 °C and 5 percent CO_2_. On the day of the permeability assay, the cells were treated with thrombin for 30 min at 2 U/mL and with 1 μg/mL FN_cf_ for 5 min. The cells were washed with ice cold PBS, fixed in 4 percent paraformaldehyde/PBS for 30 min (coverslips) or in 50 percent EtOH/PBS and 1 percent FBS for 10 min at 23 °C (MCs), and washed again with PBS. The coverslips were mounted on glass slides with Prolong Gold (Invitrogen), a DAPI-containing medium. Permeability was quantified by measuring dye (DyLight 550, Ex/Em 562⁄576 nm, ThermoFisher Scientific) fluorescence amplitude per MC in epifluorescence images at 20X magnification (EVOS FL, ThermoFisher Scientific).

### MC sorting

MAPs treated as required were fixed as above, washed twice in PBS for 10 min at 4 °C, and transferred to PBS with 1 percent FBS. MAPs were sorted at 561 nm on Biosorter (Union Biometrica) equipped with a FP-1000 nozzle.

### Expression of *dCas9-KRAB* and repressive sgRNAs in TIME cells

Lentiviri were produced and used to infect TIME cells as described^56^. The cells were transduced first by lentivirus expressing dCas9 fused to the KRAB repressor module^9^ (pHR-SFFV-dCas9-BFP-KRAB, Addgene). Lentivirus-expressing cells were sorted (FACSAria II, BD Biosciences) using blue fluorescent protein (BFP) as marker. Oligonucleotides encoding five optimized sgRNAs targeting the transcription start sites of *F2R*, *GNAQ*, and *KDR*, and NC sgRNAs were synthesized based on sequences from the CRISPRi V2 library designed by Horlbeck *et al*.^8^. Each sgRNA was subcloned into the backbone plasmid of the CRISPRi V2 library (pU6-sgRNA EF1Alpha-puro-T2A-BFP, Addgene) between the *BstXI* and *BlpI* restriction sites. Groups of *dCas9-KRAB*-expressing TIME cells were each infected separately by lentiviri expressing single sgRNAs and selected by puromycin resistance. sgRNA sequences are listed in Appendix SI, Table S1.

### Statistics

The significance of the difference between means was determined by two-tailed Student’s *t*-test. The null hypothesis was considered false if the probability satisfied the condition *P* $\le$ 0.05.

### Ethics approval and consent to participate

No animals and no humans participated in this study.

## Supplementary information

SUPPLEMENTARY INFORMATION

## Data Availability

All data and materials generated in this study will be made available to the scientific community.
