# Supplementary material for: Validation of a Miniaturized Permeability Assay Compatible with CRISPR-Mediated Genome-Wide Screen
Source: Sci Rep. 2019 Oct 2;9:14238. doi: 10.1038/s41598-019-50588-0 (PMC6775082; doi:10.1038/s41598-019-50588-0)
Supplement: Supplementary file 1 — SUPPLEMENTARY INFORMATION [file 41598_2019_50588_MOESM1_ESM.pdf]

Validation of a Miniaturized Permeability Assay Compatible with CRISPR-Mediated Genome-Wide Screen

Claire Simonneau[1§], Junning Yang[1], Xianguo Kong[1], Robert Kilker[1], Leonard Edelstein[1], Paolo Fortina[2,4], Eric Londin[3]*, and Arie Horowitz*

[1]Cardeza Center for Hematology Research, Department of Medicine, [2]Cancer Genomics and Bioinformatics Lab Department of Cancer Biology, and [3]Computational Medicine Center, Sidney Kimmel Medical College, Thomas Jefferson University, Philadelphia, Pennsylvania, USA; [4]Department of Translational and Precision Medicine, Sapienza University, Rome, Italy

*Corresponding authors

[§]Current address: Roche Innovation Center Basel, Switzerland

Corresponding authors:
Arie Horowitz; email address: arie2006@gmail.com; telephone +1-617-784-2927
Eric Londin; email address: eric.londing@jefferson.edu; telephone +1-215-503-0454

TABLE LEGEND

Table S1. Sequences of the sgRNAs from the CRISPRi V2 library used in the study, the sgRNA-specific reverse primers used in qRT-PCR experiments, and the forward primer based on the sequence of the backbone plasmid of the sgRNA library, pU6-sgRNA EF1Alpha-puro-T2A-BFP.

FIGURE LEGENDS

Figure S1: FN_{cf} identifies gaps among thrombin-treated confluent TIME cells. a. Scheme of human fibronectin showing the location of the collagen-binding domain. Numbers indicate amino acid positions. **b**. TIME cells grown on gelatin-coated glass coverslip until reaching the indicated confluences were incubated with 1 μg/mL FN_{cf} (red) for 5 min. Nuclei were stained by DAPI (blue). Scale bar, 100 μm. **c.** Confluent TIME cells grown as described above were either untreated (UT) or treated by 2 U/mL thrombin (Thr) for 30 min, followed by incubation with 1 μg/mL FN_{cf} for 5 m. **d.** Microcarriers devoid of cells (top) or carrying TIME cells at the indicated confluence (bottom) were incubated with 1 μg/mL FN_{cf} for 30 minutes. Scale bar, 200 μm.

Figure S2: The pattern of sgRNA-induced decrease in protein abundance is similar to the pattern of the decrease in gene expression. a. Densitometric quantification of PAR1 abundance in cells expressing each of the 5 *F2R*-targeting sgRNAs shown in Fig. 5a, after normalization by the weights of the corresponding β-actin loading-control bands. **b** and **c.** Densitometric quantification similar to PAR1 of $G\alpha_q$ and VEGFR2 abundances shown in Fig. 5b and 5c, respectively.

Figure S3. Full-length immunoblots of the bands shown in Fig. 5a-c. The panels to which each immunoblot corresponds are indicated.

Table S1

KDR	Protospacer	5' forward	3' reverse complement
	GCTGCCAGACGGACTTTCTG	TTGGCTGCCAGACGGACTTTCTGGTTTAAGAGC	TTAGCTCTTAAACCAGAAAGTCCGTCTGGCAGCCAACAAG
	GCGCAAGTGATGCCCGGCGC	TTGGCGCAAGTGATGCCCGGCGCGTTTAAGAGC	TTAGCTCTTAAACGCGCCGGGCATCACTTGCGCCAACAAG
	GTCTAGAGAAGGAGGCGCGG	TTGGTCTAGAGAAGGAGGCGCGGGTTTAAGAGC	TTAGCTCTTAAACCCGCGCCTCCTTCTCTAGACCAACAAG
	GAGGCAGACAGGTCGGGTGA	TTGGAGGCAGACAGGTCGGGTGAGTTTAAGAGC	TTAGCTCTTAAACTCACCCGACCTGTCTGCCTCCAACAAG
	GGCGGCGCGCAAGTGATGCC	TTGGGCGGCGCGCAAGTGATGCCGTTTAAGAGC	TTAGCTCTTAAACGGCATCACTTGCGCGCCGCCCAACAAG
SRC			
	Gggcagacggacgcacggga	TTGGggcagacggacgcacgggaGTTTAAGAGC	TTAGCTCTTAAACTCCCGTGCGTCCGTCTGCCCCAACAAG
	Ggcgggacgtccagcgggac	TTGGgcgggacgtccagcgggacGTTTAAGAGC	TTAGCTCTTAAACGTCCCGCTGGACGTCCCGCCCAACAAG
	Gcggaccgcgggacgtccag	TTGGcggaccgcgggacgtccagGTTTAAGAGC	TTAGCTCTTAAACCTGGACGTCCCGCGGTCCGCCAACAAG
	Gcaccggcagacggacgcac	TTGGcaccggcagacggacgcacGTTTAAGAGC	TTAGCTCTTAAACGTGCGTCCGTCTGCCGGTGCCAACAAG
	Gggcgggcgggcgggctcac	TTGGggcgggcgggcgggctcacGTTTAAGAGC	TTAGCTCTTAAACGTGAGCCCGCCCGCCCGCCCCAACAAG
PAR1			
	GGTGCCCACGGGTAAGATCA	TTGGGTGCCCACGGGTAAGATCAGTTTAAGAGC	TTAGCTCTTAAACTGATCTTACCCGTGGGCACCCAACAAG
	GGGACCCTGATCTTACCCGT	TTGGGGACCCTGATCTTACCCGTGTTTAAGAGC	TTAGCTCTTAAACACGGGTAAGATCAGGGTCCCCAACAAG
	GTCAGGGTCCAAGCGACCCT	TTGGTCAGGGTCCAAGCGACCCTGTTTAAGAGC	TTAGCTCTTAAACAGGGTCGCTTGGACCCTGACCAACAAG
	GTGGCTTTCGGAGGAAACTG	TTGGTGGCTTTCGGAGGAAACTGGTTTAAGAGC	TTAGCTCTTAAACCAGTTTCCTCCGAAAGCCACCAACAAG
	GTCGGCGAGCGCTGTGTCTG	TTGGTCGGCGAGCGCTGTGTCTGGTTTAAGAGC	TTAGCTCTTAAACCAGACACAGCGCTCGCCGACCAACAAG
GNAQ			
	GACTGGAGCACAGATCCggg	TTGGACTGGAGCACAGATCCgggGTTTAAGAGC	TTAGCTCTTAAACCCCGGATCTGTGCTCCAGTCCAACAAG
	GGCTCCGCGCCTGGCAATCG	TTGGGCTCCGCGCCTGGCAATCGGTTTAAGAGC	TTAGCTCTTAAACCGATTGCCAGGCGCGGAGCCCAACAAG
	GGAGCACAGATCCgggaggg	TTGGGAGCACAGATCCgggagggGTTTAAGAGC	TTAGCTCTTAAACCCCTCCCGGATCTGTGCTCCCAACAAG
	GCTCCTTCCCCGGGAACAGG	TTGGCTCCTTCCCCGGGAACAGGGTTTAAGAGC	TTAGCTCTTAAACCCTGTTCCCGGGGAAGGAGCCAACAAG
	GCGCCAGGCGCACGGCGTAG	TTGGCGCCAGGCGCACGGCGTAGGTTTAAGAGC	TTAGCTCTTAAACCTACGCCGTGCGCCTGGCGCCAACAAG
negative control			
	GCTGCATGGGGCGCGAATCA	TTGGCTGCATGGGGCGCGAATCAGTTTAAGAGC	TTAGCTCTTAAACTGATTCGCGCCCCATGCAGCCAACAAG
	GTGCACCCGGCTAGGACCGG	TTGGTGCACCCGGCTAGGACCGGGTTTAAGAGC	TTAGCTCTTAAACCCGGTCCTAGCCGGGTGCACCAACAAG
	GTGTGCAACCTCCGCCGTTG	TTGGTGTGCAACCTCCGCCGTTGGTTTAAGAGC	TTAGCTCTTAAACCAACGGCGGAGGTTGCACACCAACAAG
	GCCCGAGCAGTGGCTCGCTA	TTGGCCCGAGCAGTGGCTCGCTAGTTTAAGAGC	TTAGCTCTTAAACTAGCGAGCCACTGCTCGGGCCAACAAG
	GGTCAGGTAGAGGGATTGAG	TTGGGTCAGGTAGAGGGATTGAGGTTTAAGAGC	TTAGCTCTTAAACCTCAATCCCTCTACCTGACCCAACAAG
Forward primer	gcacaaaaggaaactcaccct		

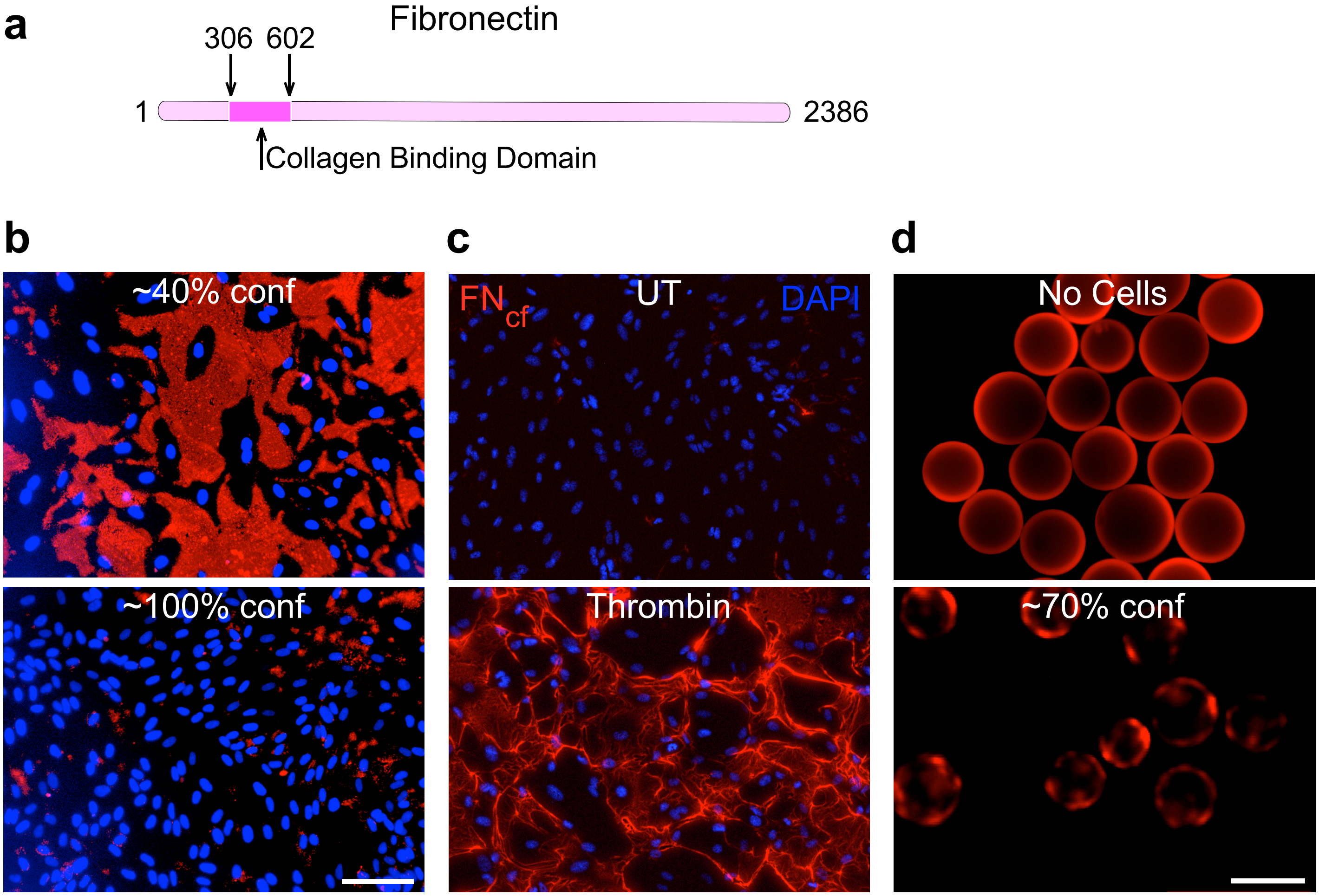
a
Fibronectin
306
602
1
2386
Collagen Binding Domain
b
~40% conf
~100% conf
c
FNcf
UT
DAPI
Thrombin
d
No Cells
~70% conf

Figure S1

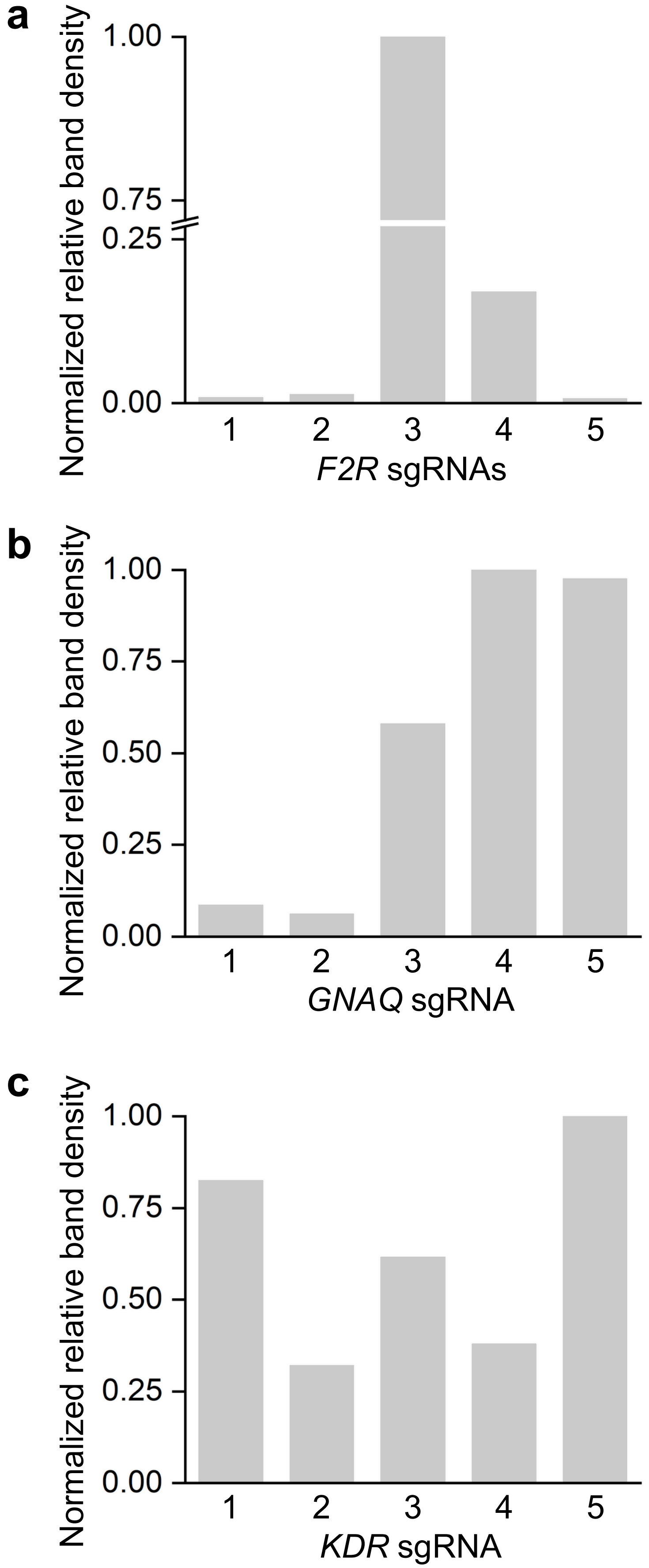
a
b
c
Normalized relative band density
1.00
0.75
0.50
0.25
0.00
1
2
3
4
5
F2R sgRNAs
GNAQ sgRNA
KDR sgRNA

Figure S2

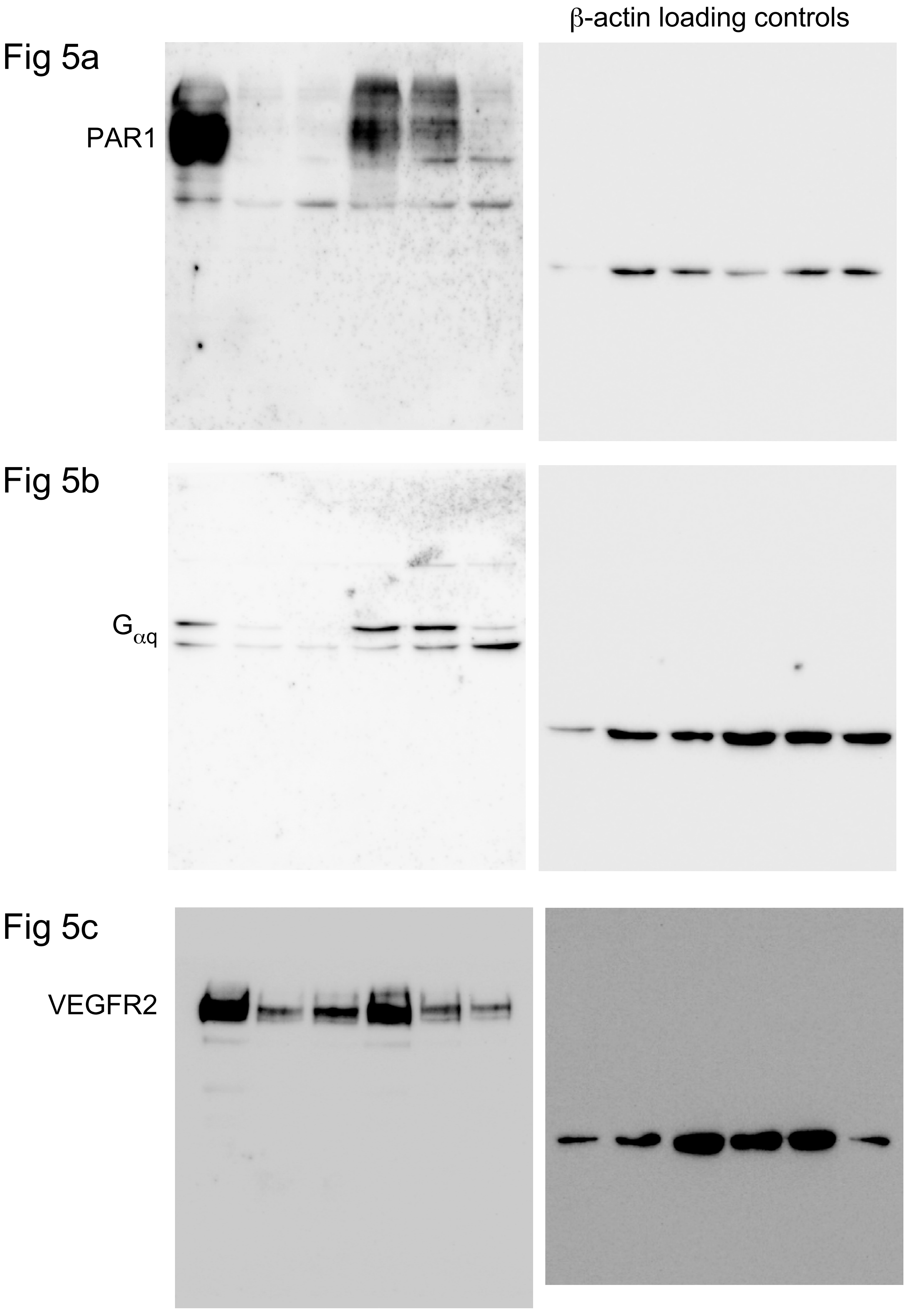

Figure S3
